# Hypoxia inducible factor-1α regulates a pro-invasive phenotype in acute monocytic leukemia

**DOI:** 10.18632/oncotarget.10660

**Published:** 2016-07-18

**Authors:** Jessica Migliavacca, Stefano Percio, Roberta Valsecchi, Elisabetta Ferrero, Antonello Spinelli, Maurilio Ponzoni, Cristina Tresoldi, Linda Pattini, Rosa Bernardi, Nadia Coltella

**Affiliations:** ^1^ Division of Experimental Oncology, IRCCS San Raffaele Scientific Institute, Milan, Italy; ^2^ Vita-Salute San Raffaele University School of Medicine, Milan, Italy; ^3^ Department of Electronics, Information and Bioengineering, Politecnico di Milano, Milan, Italy; ^4^ Department of Biomedical, Metabolic and Neural Sciences, University of Modena and Reggio Emilia, Modena, Italy; ^5^ Experimental Imaging Center, Preclinical Imaging Facility, IRCCS San Raffaele Scientific Institute, Milan, Italy; ^6^ Pathology Unit, IRCCS, San Raffaele Scientific Institute, Milan, Italy; ^7^ Unit of Hematology and Bone Marrow Transplantation, IRCCS San Raffaele Scientific Institute, Milan, Italy

**Keywords:** acute monocytic leukemia, HIF-1α, motility, invasion, self-renewal

## Abstract

Hypoxia inducible transcription factors (HIFs) are the main regulators of adaptive responses to hypoxia and are often activated in solid tumors, but their role in leukemia is less clear. In acute myeloid leukemia (AML), in particular, controversial new findings indicate that HIF-1α can act either as an oncogene or a tumor suppressor gene, and this may depend on the stage of leukemia development and/or the AML sub-type.

In this study, we find that HIF-1α promotes leukemia progression in the acute monocytic leukemia sub-type of AML through activation of an invasive phenotype. By applying a list of validated HIF-1α-target genes to different AML sub-types, we identified a HIF-1α signature that typifies acute monocytic leukemia when compared with all other AML sub-types. We validated expression of this signature in cell lines and primary cells from AML patients. Interestingly, this signature is enriched for genes that control cell motility at different levels. As a consequence, inhibiting HIF-1α impaired leukemia cell migration, chemotaxis, invasion and transendothelial migration *in vitro*, and this resulted in impaired bone marrow homing and leukemia progression *in vivo*. Our data suggest that in acute monocytic leukemia an active HIF-1α-dependent pro-invasive pathway mediates the ability of leukemic cells to migrate and invade extramedullary sites and may be targeted to reduce leukemia dissemination.

## INTRODUCTION

Acute myeloid leukemia (AML) represents almost 80% of all adult acute leukemia and is a heterogeneous disorder of the hematopoietic system caused by a number of genetic alterations and characterized by uncontrolled cell proliferation, escape from apoptosis and block of myeloid differentiation [1, 2]. The resulting growth of a clonal population of neoplastic cells in the bone marrow and blood leads to loss of normal hematopoietic functions.

Acute monocytic leukemia is the M5 sub-type of acute myeloid leukemia (AML-M5) according to the French-American-British (FAB) classification [3, 4]. AML-M5 is characterized by a differentiation arrest of the myelo-monocytic lineage at the monoblast, promonocytic or monocytic stage and constitutes 5-10% of all AML cases in adults [5, 6]. Clinically, AML-M5 is characterized by hyperleukocytosis, intravascular coagulation and a propensity to infiltrate extramedullary sites [7–9]. Beyond the phenotypic and clinical characterization, however, AML-M5 encompasses a class of genetically heterogeneous diseases with different mutations and chromosomal translocations. Among them, frequent genetic aberrations include translocations involving the *MLL* gene on chromosome 11q23, and mutations in *NPM1*, *FLT3*, *NRAS* and *DNMT3A* [10–13, 9], with *NPM1* mutations associated with favorable prognosis, and *FLT3* and *DNMT3A* mutations and *MLL* rearrangements associated with adverse prognosis [14].

Hypoxia inducible transcription factors (HIFs) are the main regulators of adaptive responses to low oxygen concentrations and are often up-regulated in solid tumors as a result of intra-tumoral hypoxia or activation of specific oncogenic pathways [15]. HIFs regulate a vast array of cellular responses in tumors, including metabolism, cell migration, invasion, metastasis and angiogenesis, and their expression often correlates with poor clinical outcome and patients survival [15–19].

In leukemia, the study of HIF factors has lagged behind for a number of years, and only recently their expression and function are beginning to be characterized. In AML in particular, a number of studies with human cells and xenograft mouse models have recently suggested that HIF-1α and HIF-2α play pro-leukemogenic functions by regulating leukemia progression and maintenance of leukemia initiating cells (LICs). As a consequence, their inhibition leads to leukemia de-bulking and eradication [20–25]. In apparent contrast with these results however, recent evidence obtained in mouse models of AML suggests that genetic deletion of *Hif-1α* or *Hif-2α* may rather promote development and/or maintenance of LICs in the presence of specific leukemogenic mutations, such as MLL rearrangements or AML1-ETO, while having no apparent effect on the progression of established leukemia [26–27]. Therefore, further characterization of the role of these factors in different AML sub-types is needed to reconcile these contrasting results and conclusively elucidate the potential of HIF inhibition for leukemia treatment.

Here, to better elucidate the involvement of hypoxia signaling in distinct AML sub-types, we applied a previously described list of *bona fide* HIF-1α target genes [24, 28] to the transcriptomic profiles of AML patients sub-categorized according to the FAB classification. We found that besides AML-M3, which we had previously identified as an AML sub-type with specific up-regulation of hypoxia signaling [24, 28], AML-M5 patients display specific up-regulation of a number of HIF-1α-target genes implicated in cell migration, invasion and transendothelial migration. In accordance with these data, inhibition of HIF-1α in a number of AML-M5 cell lines impairs leukemia motility and delays leukemia propagation *in vivo*. Therefore, our data indicate that HIF-1α exerts context-specific oncogenic functions in AML, with a pro-invasive role in AML-M5 that may be targeted to reduce leukemia dissemination.

## RESULTS

### Acute monocytic leukemia is typified by a HIF-1α-dependent signature enriched in genes promoting cell motility and invasion

We have previously reported that in acute promyelocytic leukemia (APL, AML-M3) HIF-1α inhibition delays leukemia progression and synergizes with retinoic acid in eradicating LICs [24–29]. However, recent studies have suggested that in AML HIF-1α may act as an oncogene or a tumor suppressor gene depending on the AML sub-type [30]. Here, to help elucidate the function of HIF-1α in other AML sub-types besides APL, we selected a list of validated HIF-1α-target genes [24–28] and assessed the ability of this signature to typify AML FAB sub-types.

**Figure 1:**
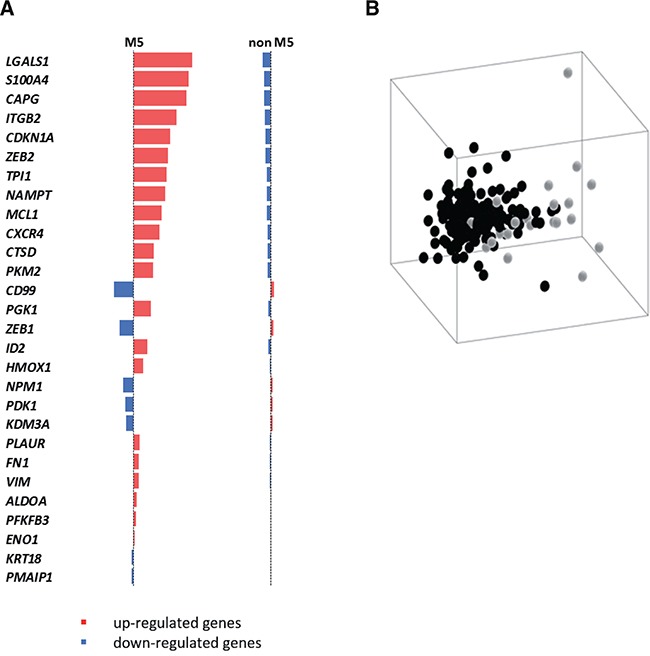
A HIF-1α sub-signature regulating cell motility acts as an AML-M5 class predictor A list of 28 genes that recognize 72% of M5 samples was selected among HIF-1α target genes using PAM algorithm **A.** Ranked list of the genes used in the classification. **B.** Multidimensional scaling on 195 AML samples using the signature obtained by the PAM classifier (M5 samples in light gray, non-M5 samples in black).

Amongst different AML, the FAB sub-type that was most significantly discriminated by Prediction Analysis of Microarray (PAM) was AML-M3, with a sensitivity of 0.800 and an accuracy of 0.964, thus confirming our previous results about the involvement of HIF-1α in APL [24–28]. Following AML-M3, PAM analysis provided the best recall for AML-M5 with a value of 0.727 (overall accuracy = 0.846), and a subset of 28 informative HIF-1α-target genes that were found most distinctive. The majority of these genes (21 out of 28, in red in Figure 1A) were found up-regulated in M5 patients compared to patients with other types of AML, while only 7 genes were down-regulated (in blue in Figure 1A). All AML samples were visualized in Figure 1B according to the expression of the 28 informative genes by multidimensional scaling.

Interestingly, the 21 HIF-1α-target genes up-regulated in AML-M5 patients were found enriched for the Gene Ontology term *cell motion* (adj. p-value = 2.40e-02), and a number of genes contained in this list are known mediators of cell migration, invasion and transendothelial migration not only in solid tumors but also in haematological malignancies (Figure 1A). LGALS1 belongs to the galectins family of beta-galactoside-binding proteins that modulate cell-cell and cell-matrix interactions, its expression correlates with tumor cell motility and invasiveness [31, 32], and is up-regulated in leukemia [33–35]. S100A4 (S100 Calcium-Binding Protein A4) is a protein involved in cell motility, invasion, and tubulin polymerization [36]; it is implicated in tumor metastasis [37, 36] and maintenance of cancer stem cells [38]. CAPG is a member of the gelsolin/villin family of actin-regulatory proteins that promotes cell migration and is over-expressed in different solid tumors [39, 40]. ITGB2 (integrin beta chain 2) regulates cell adhesion and signaling in combination with different alpha chains, and has been associated with the formation of invadosomes that facilitate leukemia cell invasion through transendothelial migration [41]. CXCR4, the receptor of stromal cell-derived factor-1 (SDF-1α), is up-regulated in different leukemic contexts and is an important regulator of chemotaxis towards protective niches in the bone marrow [42, 43].

In addition, within the top 10 genes up-regulated in AML-M5, we also found CDKN1A, which encodes a cyclin-dependent kinase inhibitor that promotes maintenance of leukemia stem cells [44], and the ZEB2 transcription factor, a master regulator of epithelial to mesenchymal transition [45] that also has been implicated in cancer and leukemia stem cell maintenance [46–48].

**Figure 2:**
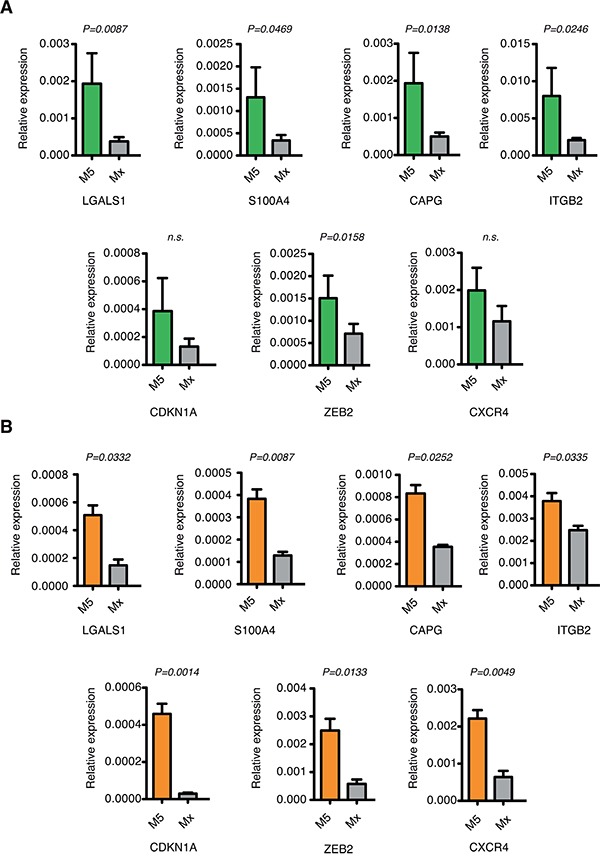
Validation of the top up-regulated HIF-1α-target genes in AML-M5 cells **A.** Real-time PCR analysis of LGALS1, S100A4, CAPG, ITGB2, CDKN1A, ZEB2 and CXCR4 in primary bone marrow samples from M5 (n=7) and other FAB sub-types (n=17) AML patients. Data are represented as mean values ± s.e.m. **B.** Real-time PCR analysis of LGALS1, S100A4, CAPG, ITGB2, CDKN1A, ZEB2 and CXCR4 in human M5 cell lines (n=3) and other AML cell lines (n=4). Data are represented as mean values ± s.e.m of three independent experiments.

In order to validate the data obtained by *in silico* analysis, we measured the expression of the top up-regulated HIF-1α-target genes in primary leukemic bone marrow samples from AML patients diagnosed and treated at San Raffaele Hospital. Real time PCR analysis confirmed up-regulation of LGALS1, S100A4, CAPG, ITGB2, CDKN1A, ZEB2 and CXCR4 in AML-M5 patients compared with other AML FAB sub-types (Figure 2A).

To corroborate these findings in cell models more amenable to *in vitro* manipulation we analyzed expression of LGALS1, S100A4, CAPG, ITGB2, CDKN1A, ZEB2 and CXCR4 in the AML cell lines: KG-1, Kasumi-1, HL-60 and NB4 representative of FAB sub-types from M0 to M3, and the M5 cell lines MOLM-13, THP-1 and Mono Mac 6. Expression analysis confirmed up-regulation of all genes in cell lines of the M5 FAB sub-type compared with other cell lines (Figure 2B).

**Figure 3:**
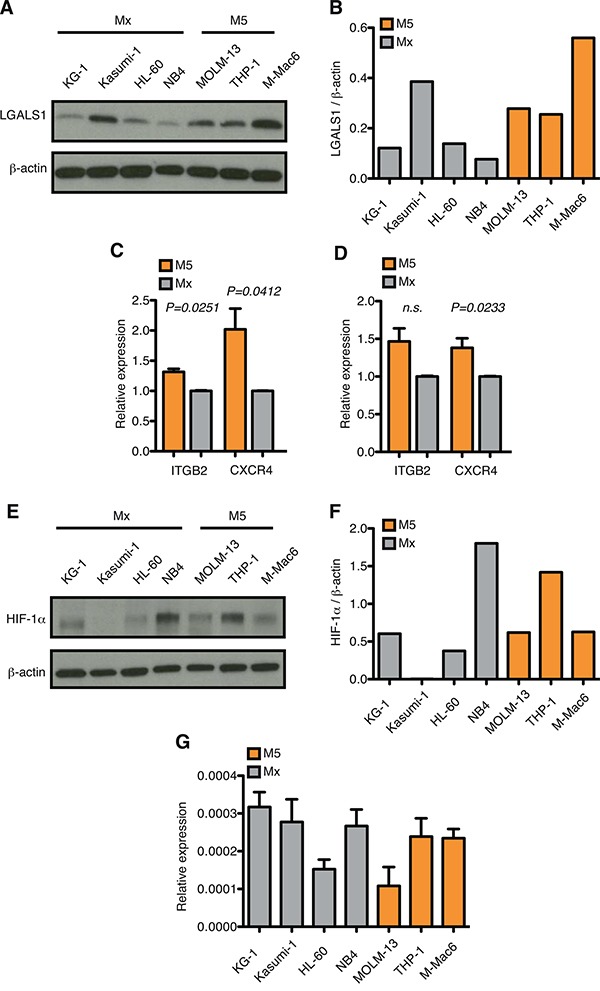
HIF-1α protein expression is up-regulated in AML-M5 cell lines **A.** Immunoblot of LGALS1 in human M5 cell lines (n=3) and other AML cell lines (n=4). **B.** Quantification of immunoblot in (A). Graph represents the ratio between LGALS1 over β-actin. **C.** Flow cytometric analysis of ITGB2 and CXCR4 positive cells (percentage of + cells). Graph represents the relative expression of ITGB2 and CXCR4 in human M5 cell lines over other AML cell lines. Data represent mean values ± s.e.m of three independent experiments. **D.** MFI (mean fluorescent intensity) of ITGB2 and CXCR4 in human M5 cell lines over other AML cell lines. Data represent mean values ± s.e.m of three independent experiments. **E.** Immunoblot of HIF-1α in human M5 cell lines (n=3) and other AML cell lines (n=4). **F.** Quantification of immunoblot in (E). Graph represents the ratio between HIF-1α over β-actin. **G.** Real-time PCR analysis of HIF-1α in human AML cell lines. Data are represented as mean values ± s.e.m of three independent experiments.

In agreement with mRNA data, increased expression of selected genes, such as LGALS1, ITGB2 and CXCR4 was observed in AML-M5 cell lines also at the protein level, either by western blotting or surface expression (Figure 3A–3D). Interestingly, we observed that the HIF-1α protein is expressed in normoxic conditions in a number of AML cell lines, with highest expression in NB4 cells, representative of AML-M3, followed by M5 cell lines (Figure 3E and 3F). These differences are not mirrored by corresponding mRNA levels (Figure 3G), thus suggesting that HIF-1α is up-regulated through post-transcriptional mechanisms yet to be identified in AML cells of different origin.

Taken together, these results indicate that in the M5 sub-type of AML a HIF-1α-dependent signature that regulates migration of leukemic cells is up-regulated as compared to other types of AML.

### HIF-1α silencing impairs cell migration and invasion in AML-M5 leukemic cells

**Figure 4:**
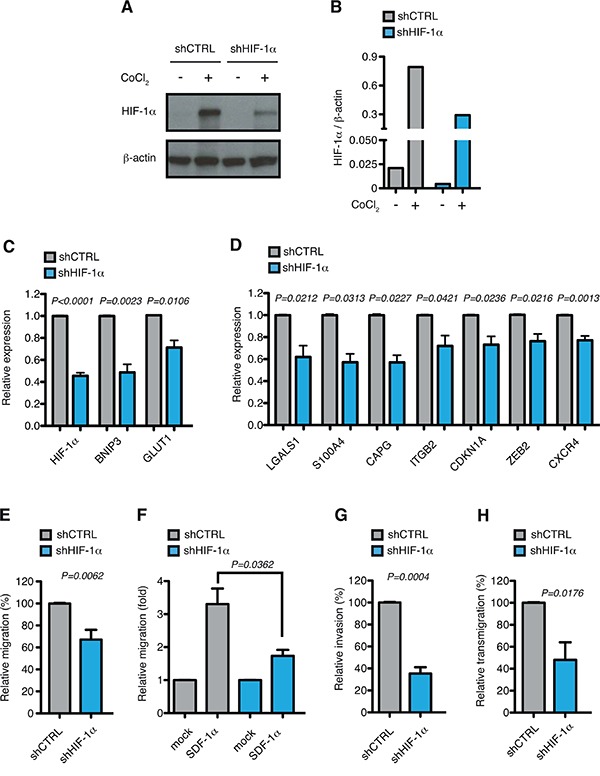
HIF-1α chronic silencing impairs motility of MOLM-13 cells *in vitro* **A.** Immunoblot of HIF-1α in MOLM-13 cells transduced with lentiviral vectors carrying control (shCTRL) or HIF-1α-directed shRNA (shHIF-1α). When indicated shCTRL and shHIF-1α MOLM-13 cells were treated with CoCl_2_ to increase HIF-1α stability. **B.** Quantification of immunoblot in (A). Graph represents the ratio between HIF-1α over β-actin. **C.** Real-time PCR analysis of HIF-1α and its target genes BNIP3 and GLUT1 in MOLM-13 cells transduced as in (A). Data represent mean values ± s.e.m. of three independent experiments. **D.** Real-time PCR analysis of LGALS1, S100A4, CAPG, ITGB2, CDKN1A, ZEB2 and CXCR4 in shHIF-1α MOLM-13 cells relative to shCTRL cells. Data represent mean values ± s.e.m. of three independent experiments. **E.** Basal migration, expressed as percentage of shHIF-1α MOLM-13 cells relative to shCTRL. Data represent mean values ± s.e.m. of three independent experiments. **F.** SDF-1α induced migration (fold increase) of shCTRL and shHIF-1α MOLM-13 cells relative to basal migration of their respective control. Data represent mean values ± s.e.m. of three independent experiments. **G.** Basal invasion, expressed as percentage of shHIF-1α MOLM-13 cells relative to shCTRL. Data represent mean values ± s.e.m. of three independent experiments. **H.** Transendothelial migration through a HUVEC endothelial layer, expressed as percentage of shHIF-1α MOLM-13 cells relative to shCTRL. Data represent mean values ± s.e.m. of three independent experiments.

To evaluate the function of HIF-1α towards controlling the expression of genes involved in cell motion in AML-M5, HIF-1α was stably down-regulated in MOLM-13 cells using validated shRNAs [24, 49]. HIF-1α silencing led to about 50% reduction in HIF-1α expression at mRNA and protein level (Figure 4A–4C). Accordingly, the expression of common HIF-1α-target genes like BNIP3 and GLUT1 was significantly reduced upon HIF-1α silencing (Figure 4C). In addition, the top up-regulated HIF-1α-target genes in AML-M5 were all down-regulated after HIF-1α inhibition (Figure 4D), thus indicating that also in the context of AML-M5 these genes are regulated by HIF-1α.

We next tested the functional consequences of HIF-1α down-regulation. Proliferation rates and basal apoptosis were not affected by chronic HIF-1α silencing in MOLM-13 cells (Supplementary Figure 1A and B). As HIF-1α inhibition led to down-regulation of genes involved in migration, chemotaxis, invasion and transendothelial migration, the motile and invasive phenotypes of MOLM-13 cells were tested after HIF-1α silencing. Both basal and SDF-1α-induced cell migration were impaired in HIF-1α down-modulated cells (Figure 4E and 4F), according with CXCR4 inhibition. In addition, cell invasion through matrigel and transendothelial migration through a HUVEC monolayer were also significantly impaired upon reduced HIF-1α expression (Figure 4G and 4H), consistently with down-regulation of LGALS1, S100A4 and ITGB2 that mediate invasive and infiltrating phenotypes.

**Figure 5:**
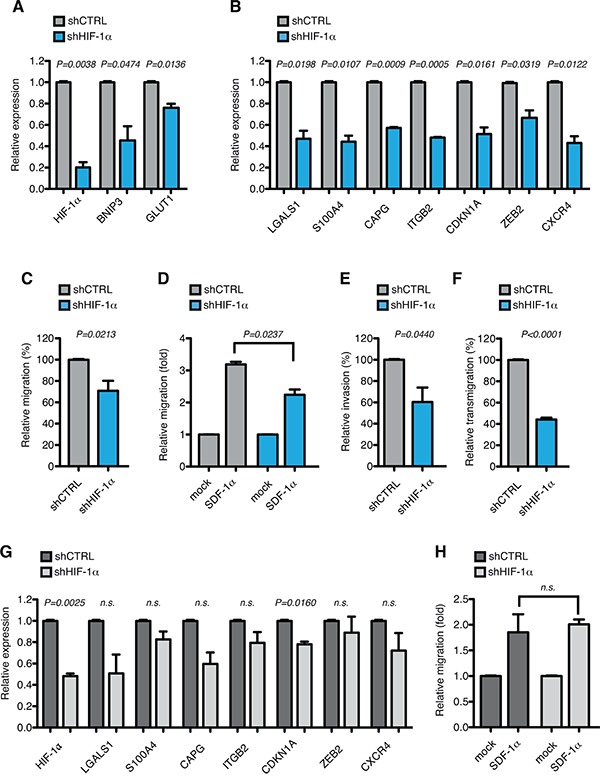
HIF-1α chronic silencing impairs motility of THP-1 cells *in vitro* **A.** Real-time PCR analysis of HIF-1α and its target genes BNIP3 and GLUT1 in THP-1 cells transduced with lentiviral vectors carrying control (shCTRL) or HIF-1α-directed shRNA (shHIF-1α). Data represent mean values ± s.e.m. of three independent experiments. **B.** Real-time PCR analysis of LGALS1, S100A4, CAPG, ITGB2, CDKN1A, ZEB2 and CXCR4 in shHIF-1α THP-1 cells relative to shCTRL cells. Data represent mean values ± s.e.m. of three independent experiments. **C.** Basal migration, expressed as percentage of shHIF-1α THP-1 cells relative to shCTRL. Data represent mean values ± s.e.m. of three independent experiments. **D.** SDF-1α induced migration (fold increase) of shCTRL and shHIF-1α THP-1 cells relative to basal migration of their respective control. Data represent mean values ± s.e.m. of three independent experiments. **E.** Basal invasion, expressed as percentage of shHIF-1α THP-1 cells relative to shCTRL. Data represent mean values ± s.e.m. of three independent experiments. **F.** Transendothelial migration through a HUVEC endothelial layer, expressed as percentage of shHIF-1α THP-1 cells relative to shCTRL. Data represent mean values ± s.e.m. of three independent experiments. **G.** Real-time PCR analysis of HIF-1α, LGALS1, S100A4, CAPG, ITGB2, CDKN1A, ZEB2 and CXCR4 in Kasumi-1 cells transduced with lentiviral vectors carrying control (shCTRL) or HIF-1α-directed shRNA (shHIF-1α). Data represent mean values ± s.e.m. of three independent experiments. **H.** SDF-1α induced migration (fold increase) of shCTRL and shHIF-1α Kasumi-1 cells relative to basal migration of their respective control. Data represent mean values ± s.e.m. of three independent experiments.

To substantiate our data in another relevant context, we inhibited HIF-1α in a second AML-M5 cell line, THP-1. Similarly to MOLM-13 cells, down-regulation of HIF-1α and common target genes BNIP3 and GLUT1 (Figure 5A) was accompanied by inhibition of HIF-1α-target genes involved in migration, invasion and self-renewal in THP-1 cells (Figure 5B). Accordingly, HIF-1α silencing resulted in inhibition of basal migration, SDF-1α-mediated chemotaxis, cell invasion and transendothelial migration (Figure 5C–5F). To understand if regulation of the motile phenotype by HIF-1α occurs specifically in AML-M5, we stably silenced HIF-1α in a cell line representative of AML-M2 (Kasumi-1 cells). Interestingly, although as expected also in this cell line HIF-1α down-regulation led to inhibition of the target genes implicated in cell motility, albeit more modestly (Figure 5G), basal and SDF-1α-mediated cell migration were not affected by HIF-1α silencing (Figure 5H and data not shown). This indicates that the function of HIF-1α towards inducing cell motility may be particularly relevant in AML-M5, which is consistent with the evidence that the genes that we have analyzed are expressed at higher levels in this AML sub-type (Figure 2 and 3).

Taken together, these data indicate that HIF-1α activates a pro-invasive gene signature in acute monocytic leukemia that leads to increased motility, invasion and transendothelial migration, and inhibition of HIF-1α impairs important functions of monocytic leukemia blasts.

### Acute HIF-1α silencing recapitulates HIF-1α chronic silencing

To corroborate the findings obtained with HIF-1α chronic silencing, and to establish whether more pronounced and acute inhibition of HIF-1α may further impinge on the functions of AML-M5 leukemic cells, we next used the RNA antagonist EZN-2968, a locked nucleic acid-modified oligonucleotide (LNA-ON) targeting HIF-1α, and its control LNA-ON EZN-3088 [50].

**Figure 6:**
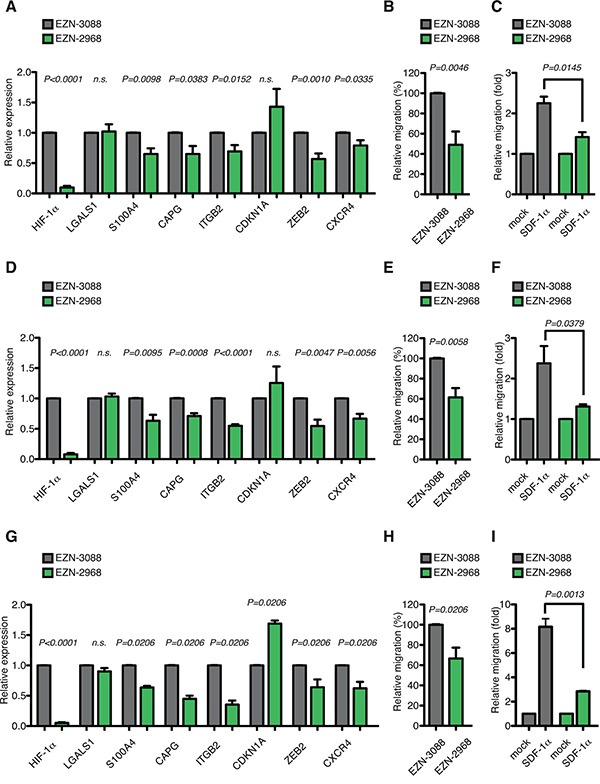
Acute HIF-1α silencing recapitulates HIF-1α chronic silencing in different AML-M5 cell lines *in vitro* **A.** Real-time PCR analysis of HIF-1α, LGALS1, S100A4, CAPG, ITGB2, CDKN1A, ZEB2 and CXCR4 in MOLM-13 cells 24 h after transfection with EZN-2968 relative to cells transfected with EZN-3088. Data represent mean values ± s.e.m. of three independent experiments. **B.** Basal migration, expressed as percentage of MOLM-13 cells transfected with EZN-2968 relative to cells transfected with EZN-3088. Data represent mean values ± s.e.m. of three independent experiments. **C.** SDF-1α induced migration (fold increase) of EZN-3088 and EZN-2968 transfected MOLM-13 cells, relative to basal migration of their respective control. Data represent mean values ± s.e.m. of three independent experiments. **D.** Real-time PCR analysis of HIF-1α, LGALS1, S100A4, CAPG, ITGB2, CDKN1A, ZEB2 and CXCR4 in THP-1 cells 24 h after transfection with EZN-2968 relative to cells transfected with EZN-3088. Data represent mean values ± s.e.m. of three independent experiments. **E.** Basal migration, expressed as percentage of THP-1 cells transfected with EZN-2968 relative to cells transfected with EZN-3088. Data represent mean values ± s.e.m. of three independent experiments. **F.** SDF-1α induced migration (fold increase) of EZN-3088 and EZN-2968 transfected THP-1 cells, relative to basal migration of their respective control. Data represent mean values ± s.e.m. of three independent experiments. **G.** Real-time PCR analysis of HIF-1α, LGALS1, S100A4, CAPG, ITGB2, CDKN1A, ZEB2 and CXCR4 in Mono Mac 6 cells 24 h after transfection with EZN-2968 relative to cells transfected with EZN-3088. Data represent mean values ± s.e.m. of three independent experiments. **H.** Basal migration, expressed as percentage of Mono Mac 6 cells transfected with EZN-2968 relative to cells transfected with EZN-3088. Data represent mean values ± s.e.m. of three independent experiments. **I.** SDF-1α induced migration (fold increase) of EZN-3088 and EZN-2968 transfected Mono Mac 6 cells, relative to basal migration of their respective control. Data represent mean values ± s.e.m. of three independent experiments.

EZN-2968 transfection led to a reduction of more than 90% of HIF-1α mRNA levels in AML-M5 MOLM-13, THP-1 and Mono Mac 6 cells (Figure 6A, 6D and 6G). In accordance with the data previously obtained upon stable silencing of HIF-1α (Figure 4D and 5B), acute HIF-1α silencing resulted in similar down-regulation of the top AML-M5 HIF-1α-target genes in the three cells lines, with the notable exception of LGALS1 and CDKN1A, which were consistently found not regulated or up-regulated respectively (Figure 6A, 6D and 6G). While the lack of LGALS1 regulation in conditions of acute HIF-1α silencing may indicate that LGALS1 is a late-response gene, or that it is indirectly regulated by HIF-1α in this context, CDKN1A up-regulation correlated with decreased proliferation and increased apoptosis when HIF-1α was strongly inhibited, which was not observed upon milder and chronic HIF-1α silencing (please, compare Supplementary Figure 1A and B with Supplementary Figure 3B and C). As HIF-1α is an essential regulator of anaerobic glycolysis, AML-M5 neoplastic cells may be addicted to minimal levels of HIF-1α expression for proliferation and survival, and acute and strong HIF-1α inhibition obtained with EZN-2968 may be detrimental to the cells. Importantly, EZN-2968 also impaired cell migration, both in basal conditions and upon SDF-1α stimulation, in all AML-M5 cell lines tested (Figure 6B, 6C, 6E, 6F, 6H and 6I).

Taken together these data indicate that acute silencing of HIF-1α impairs important pro-leukemogenic functions like basal cell migration and chemotaxis of AML-M5 cells *in vitro* and may therefore result in impaired leukemia progression or propagation *in vivo*.

### HIF-1α inhibition results in impaired leukemia homing and development in a xenograft model of acute monocytic leukemia *in vivo*

To understand whether HIF-1α inhibition led to impaired leukemia development and progression *in vivo* we took advantage of a MOLM-13 xenograft model of acute monocytic leukemia that mimics human leukemia in that mice develop a rapid and fatal acute myeloid leukemia localizing to the bone marrow, spleen and peripheral blood upon intravenous injections [51].

**Figure 7:**
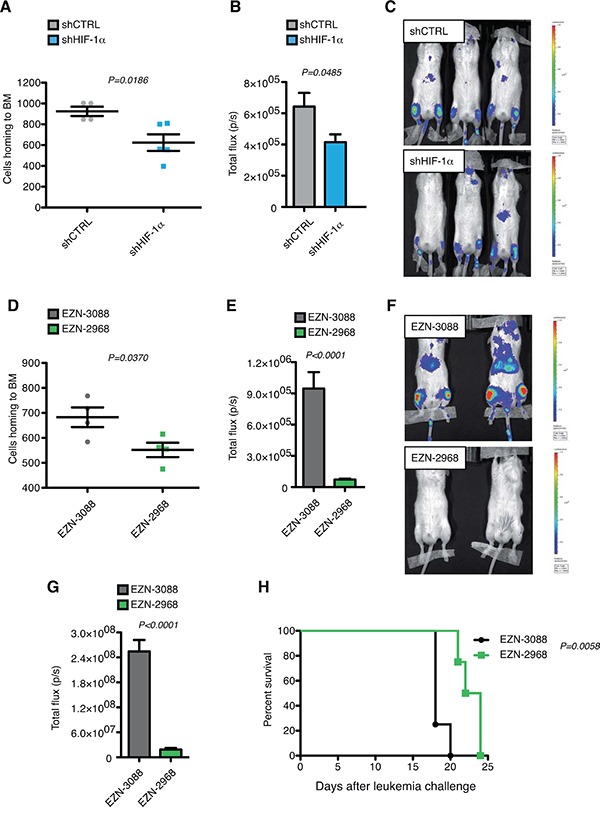
HIF-1α inhibition impairs leukemia homing and progression in MOLM-13 xenograft model *in vivo* **A.** Total numbers of human ΔNGFR^+^ shCTRL or shHIF-1α MOLM-13 cells localizing to the bone marrow (BM) of NSG mice 16 h after tail vein injection (over 5 × 10^6^ events; n = 4 for shCTRL, and n=5 shHIF-1α). **B.** Quantified light output expressed as total flux (photons/seconds) from each ROI drawn on the right and left posterior legs of mice injected with MOLM-13 shCTRL and shHIF-1α and co-expressing ΔNGFR and luciferase 5 days post leukemia challenge (n=3 for each group) **C.** Bioluminescence images of mice described in (B). All images are plotted with the same scale. **D.** Total numbers of human ΔNGFR^+^ MOLM-13 cells transfected with EZN-3088 or EZN-2988 and localizing to the BM of NSG mice 16 h after tail vein injection (over 5 × 10^6^ events; n=4 for each group). Cells were injected 24 h after transfection. **E.** Quantified light output expressed as total flux (photons/seconds) from each ROI drawn on the right and left posterior legs of mice injected with MOLM-13 transfected with EZN-3088 or EZN-2988 and co-expressing ΔNGFR and luciferase 5 days post leukemia challenge (n=4 for each group). **F.** Bioluminescence images of representative mice described in (E). **G.** Quantified light output expressed as total flux (photons/seconds) from each ROI drawn on the right and left posterior legs of mice injected with MOLM-13 transfected with EZN-3088 or EZN-2988 and co-expressing ΔNGFR and luciferase 9 days post leukemia challenge (n=4 for each group). **H.** Kaplan-Meier survival curve of NSG mice injected with MOLM-13 transfected with EZN-3088 or EZN-2988 and co-expressing ΔNGFR and luciferase (n=4 for each group). Survival curves were analyzed with the Mantel-Cox test.

MOLM-13 cells, transduced with shRNA constructs (shCTRL and shHIF-1α) and a lentiviral vector co-expressing luciferase and ΔNGFR, were transplanted intravenously into immunocompromised NSG mice. Consistently with down-modulation of CXCR4 (Figure 4D), cells with reduced expression of HIF-1α showed defective bone marrow homing 16 hours after injection (Figure 7A). As a consequence, short-term colonization of the bone marrow was still impaired 5 days after injection (Figure 7B and 7C). However, this difference was progressively lost at later time points, and when animals were sacrificed because terminally sick no difference in leukemia involvement was observed in various organs (Supplementary Figure 2A-2C). Accordingly, we did not detect any difference in terms of survival of transplanted animals (data not shown). However, real time PCR analysis of leukemic bone marrow at the end of the experiment revealed that HIF-1α silencing was lost *in vivo* (Supplementary Figure 2D). Although sorted for GFP expression, injected cells comprised a multi-clonal population of cells expressing different levels of GFP (and presumably HIF-1α); therefore, it is likely that loss of HIF-1α silencing reflects a counter selection of cells with lower HIF-1α levels under increased *in vivo* selective pressure.

To establish whether stronger and acute HIF-1α silencing may impact more profoundly on leukemia onset and progression, luciferase and ΔNGFR co-expressing MOLM-13 cells were electroporated with EZN-2968 and its control oligonucleotide EZN-3088 and injected into NSG mice. Interestingly, acute HIF-1α silencing led to decreased proliferation and increased apoptosis 24 hours after *in vitro* transfection (Supplementary Figure 3B and 3C), which was not observed upon chronic and milder inhibition (Supplementary Figure 1). Similar to chronic HIF-1α inhibition, acute silencing also resulted in impaired bone marrow homing (Figure 7D). In addition however, acute silencing of HIF-1α led to prolonged inhibition of bone marrow colonization, as measured by *in vivo* luciferase activity at day 5 and day 9 from leukemia challenge (Figure 7E–7G), and prolonged mice survival (Figure 7H). To better elucidate the effect of HIF-1α acute silencing on the dissemination of leukemic cells and leukemia colonization *in vivo*, leukemia involvement was analyzed in different organs at different time points. 9 days post leukemia challenge a significant percentage of MOLM-13 cells transfected with control LNA-ON was found in bone marrow and spleen of NSG mice, and a smaller percentage also colonized liver and lungs (Supplementary Figure 3D). HIF-1α silenced cells were in general less prone to engraft and colonize not only bone marrow and spleen but also all other organs (Supplementary Figure 3D). This difference was still maintained 15 days post-leukemia challenge (Supplementary Figure 3E), while it was lost when mice were sacrificed because terminally sick (Supplementary Figure 3F-H). Also, similar to what was observed upon chronic HIF-1α down-regulation, HIF-1α inhibition in bone marrow leukemic cells was lost at the end of experiment upon acute HIF-1α silencing (Supplementary Figure 3I), perhaps less surprisingly as oligonucleotide-mediated inhibition may be lost upon cell division, and cells with strong HIF-1α inhibition were found to be prone to cell cycle arrest and apoptosis *in vitro* (Supplementary Figure 3B and 3C).

Taken together these data show that hampering HIF-1α expression in a mouse model of AML-M5 results in impairment of bone marrow homing, colonization and leukemia progression, thus indicating that in this AML context HIF-1α plays oncogenic functions.

## DISCUSSION

HIF-1α is a master regulator of cellular responses to low oxygen concentrations in both physiological and pathological conditions. The role of HIF-1α has been extensively studied in solid tumors, where often it has been found up-regulated due to intratumoral hypoxia or oncogene signaling, and where it regulates a vast array of pro-tumoral responses including metabolism, cell survival, neo-angiogenesis, invasion and metastasis [15]. For this reason, a number of compounds inhibiting HIF-1α are under development for treating solid tumors [16]. Nonetheless, additional evidence indicates that in specific tumor contexts such as in renal cancer HIF-1α may also play tumor suppressive functions [17], thus indicating that the clinical application of pre-clinical studies with HIF inhibitors should be carefully planned.

The role of HIF-1α in leukemia, and in particular in acute myeloid leukemia, is only recently beginning to be characterized and at the present time it is highly debated. HIF-1α was first described as highly expressed in the LICs compartment of human AML, identified as CD34^+^CD38^-^ cells, albeit no distinction was made between different sub-types of AML [20]. By using an inhibitor of HIF-1α, it was suggested that HIF-1α is a key regulator of leukemia stem cell maintenance in AML [20]. A bias of this study however is that LICs were analyzed only in the CD34^+^CD38^-^ fraction, which is not present in all AML, thus suggesting that the role of HIF-1α on LICs maintenance should be tested more thoroughly in different cell types and different patients, especially those with low CD34 expression [30].

After these first indications, we described a pro-leukemogenic function of HIF-1α in a specific sub-type of AML, that is acute promyelocytic leukemia or AML-M3, due to a specific functional cooperation of HIF-1α with the oncogenic fusion protein PML-RARα [24]. In this context, we demonstrated that HIF-1α regulates the clonogenicity of leukemic cells, as a read-out of leukemia-initiating capacity, but also other pro-leukemogenic functions such as bone marrow neo-angiogenesis, chemotaxis and leukemia migration/dissemination [24]. Interestingly, a study published in the same year challenged the concept that HIF-1α promotes leukemogenesis in AML [26]. More specifically, genetic deletion of *Hif-1α* before leukemia initiation by different AML oncogenic mutations (AML1-ETO, MLL-AF9, HOXA9/MEIS1) revealed that loss of Hif-1α expression did not delay leukemia initiation or progression in any of the genetic models tested, and, contrary to previous data, its deletion either did not regulate or rather increased leukemia self-renewal in secondary transplantation experiments [26]. This study however did not address the role of HIF-1α in established human leukemia carrying the same oncogenic mutations.

Taken together, these studies present an intricate scenario where the role of HIF-1α in AML is still controversial and it may change at different stages of leukemia development and/or in specific leukemia contexts.

With our current work, by undertaking an *in silico* approach aimed to identify AML patients with deregulated hypoxia signaling, we found that besides AML-M3, where we had previously implicated HIF factors [24, 28, 29], patients with the M5 sub-type of AML also show significant up-regulation of HIF-1α-target genes as compared to all other AML sub-types. Interestingly, a number of genes regulating cell migration were found up-regulated in AML-M5, which is consistent with the propensity of cells of this AML sub-type to infiltrate extramedullary sites [7, 8]. Higher expression of these genes was validated in primary AML-M5 cells and in cell lines, as well as their dependency on HIF-1α expression. Interestingly, we observed that within the AML cell lines that we analyzed, AML-M3 and AML-M5 cells express high levels of HIF-1α protein even in normoxic conditions, and this up-regulation does not occur at the transcriptional level. Although further studies are needed to better understand the molecular basis of this regulation, these data are in line with our previous and current results showing that HIF-1α is importantly implicated in AML-M3 and AML-M5 pathogenesis [24].

In validation of the gene expression results, we found that chronic and acute suppression of HIF-1α impaired chemotaxis, cell motility and cell invasion in cell lines representative of AML-M5. This resulted in impaired bone marrow homing and colonization *in vivo*, together with impaired dissemination and engraftment of leukemic cells to different organs. The long-term effects of HIF-1α suppression *in vivo* were particularly evident upon acute and strong HIF-1α inhibition with a specific oligonucleotide. However, as in these circumstances we also observed decreased proliferation and increased apoptosis compared to chronic and stable HIF-1α silencing, we hypothesize that ongoing growth arrest and cell death may have contributed to decreased engraftment and *in vivo* leukemia colonization, besides defective cell migration. Nonetheless mice survival was only modestly prolonged upon acute HIF-1α silencing, as AML-M5 cells reacquired HIF-1α expression *in vivo*, both upon acute and chronic HIF-1α silencing, thus indicating that cells with reduced HIF-1α expression were being counter selected possibly due to increased *in vivo* selective pressure.

Taken together, these results indicate that in AML-M5 HIF-1α plays specific pro-oncogenic functions that are mainly related to promoting leukemia cell motility and dissemination, but robust HIF-1α suppression may exert anti-leukemic functions also by inducing cell death and blasts eradication.

Although the M5 sub-type of AML encompasses a class of leukemia with different genetic abnormalities, including mutations associated with favorable prognosis (*NPM1)*, and mutations or chromosomal aberrations associated with adverse prognosis (*FLT3*, *DNMT3A, MLL* fusions*)* [14], we find that HIF-1α exerts similar effects in cell lines representative of genetically different AML-M5, as it promotes migration and invasion both in MOLM-13 cells characterized by FLT3 mutation, and THP-1 cells, which harbor an MLL-AF9 translocation (www.dsmz.de). Also, we found that patients representative of different genetic sub-groups within AML-M5 shared the same up-regulation of HIF-1α responsive genes irrespective of their mutational status (data not shown). Interestingly, we did not find any up-regulation of the AML-M5 hypoxia sub-signature in M5 leukemic blasts when compared to normal monocytes, thus suggesting that up-regulation of this specific HIF-1α sub-signature may be a feature of monocytes, which is amplified in a leukemic setting (data not shown). Taken together, these results suggest that differently from AML-M3, where HIF-1α specifically cooperates with the fusion protein PML-RARα, in AML-M5 HIF-1α may regulate the expression of a set of genes that are specifically expressed in monocytic progenitors, independently from the genetic insult that led to their oncogenic transformation.

In conclusion, our studies demonstrate that HIF-1α exerts oncogenic functions in AML-M5 and prompt future examination on the role of compounds that inhibit HIF factors in combination with other therapies for the treatment of acute monocytic leukemia.

## MATERIALS AND METHODS

### AML gene expression data set

We retrieved gene expression profiles from The Cancer Genome Atlas (TCGA) Data Portal, (https://tcga-data.nci.nih.gov/tcga/tcgaHome2.jsp; accession LAML, level 3 (gene level) correspondent to samples of bone marrow tissue from AML patients analyzed using the Affymetrix Human Genome U133 Plus 2.0 Array; we considered the samples with explicit French-American-British (FAB) classification. We analyzed a total of 195 AML patients divided from M0 to M7 according to the FAB classification. The samples were divided as follows: M0 n=17, M1 n=44, M2 n=44, M3 n=20, M4 n=42, M5 n=22, M6 n=3 and M7 n=3.

### Gene expression analysis

To investigate the contribution of hypoxia-related signaling in acute monocytic leukemia (M5) compared to other AML sub-types, we used a previously described list of *bona fide* HIF-1α target genes [28] and assessed the capability of this signature to discriminate M5 from other AML FAB sub-types based on gene expression profiles. For this purpose, we applied a supervised approach, the Prediction Analysis of Microarray (PAM) [52] available in the R environment, in which the nearest shrunken centroids classifier is used to evaluate the accuracy of the classification and to identify genes whose expressions is most distinctive for the M5-class. The complete data set of AML samples was visualized according to the selected signature in a 3D space of multidimensional scaling (MDS) plot using the standard euclidean metric as the measure of dissimilarity. The annotation enrichment analysis was performed using the David Function Annotation tool [53].

### Cell lines and reagents

The human leukemic cell lines KG-1, Kasumi-1, HL-60, NB4, MOLM-13, THP-1 and Mono Mac 6 were maintained in RPMI 1640 and HEK-293T cells in IMDM media supplemented with 10% FBS and standard antibiotics (Lonza). Primary human umbilical vein endothelial cells (HUVEC) were obtained and cultured as previously described [54]. All cells were maintained at 37°C in a humidified atmosphere containing 5% CO_2_.

EZN-3088 (control LNA-ON for HIF-1α) and EZN-2968 (LNA-ON for HIF-1α) [50] were provided by Belrose Pharma Inc. and used in accordance with the manufacturer's instructions. MOLM-13, THP-1 and Mono Mac 6 cells were transfected with EZN-3088 and EZN-2968 in an Amaxa™ Nucleofector™ System (Lonza). CoCl_2_, Trypan blue and puromycin were purchased from Sigma; SDF-1α (CXCL12) was from Peprotech.

### Patients' samples

Bone marrow samples from AML patients of different leukemia sub-type according to the FAB classification and containing > 60% primary leukemia blasts were collected upon written informed consent in accordance with the Declaration of Helsinki by the Hematology and Bone Marrow Transplantation Unit at IRCCS Ospedale San Raffaele and stored at OSR AML Bio Bank. This study was approved by the Institutional Review Boards of San Raffaele Scientific Institute, Milan.

### Lentiviral vectors

GIPZ HIF-1α or control shRNA plasmids were from Open Biosystems. Lentiviral vectors were obtained by HEK-293T transfection as previously described [55]. MOLM-13, THP-1 and Kasumi-1 cells were transduced by spinoculation, selected with puromycin (4 μg/mL, 7 μg/mL and 1 μg/mL respectively) and sorted for GFP expression 2 weeks after transduction (MoFlo XDP, Beckman Coulter), leading to a bulk of cells with different integrations and GFP levels.

For *in vivo* experiments, shCTRL and shHIF-1α MOLM-13 cells were further transduced with a bidirectional lentiviral vector co-expressing luciferase and truncated nerve growth factor receptor (ΔNGFR) (kindly provided by G. Escobar, B. Gentner and L. Naldini, unpublished data) and sorted for ΔNGFR (CD271, PE, BD Pharmingen) expression 2 weeks after transduction.

For *in vivo* experiments with LNA-ON (EZN-3088 and EZN-2968), MOLM-13 cells transduced with the bidirectional lentiviral vector co-expressing luciferase and ΔNGFR and sorted for ΔNGFR expression 2 weeks after transduction, were electroporated with EZN-3088 and EZN-2968 and injected in mice after 24 hours.

### Real-time PCR

RNA was isolated with the RNeasy mini kit (Qiagen) and cDNA was obtained by retro-transcription of 1 μg total RNA using Advantage RT for PCR kit (Clontech) and analyzed by real-time PCR in 7900 Fast Real-Time PCR System (Applied Biosystem). All probes for TaqMan assays were purchased from Applied Biosystem. 18S was used as internal control.

The relative expression of different cDNAs was calculated using the 2^−ΔΔCt^ method with respect to control conditions, except for assessing the relative expression of LGALS1, S100A4, CAPG, ITGB2, CDKN1A, ZEB2 and CXCR4 in primary AML samples and cell lines in Figure 2 and HIF-1α in Figure 3, which were calculated by the 2^−ΔCt^ method relative to 18S expression.

### Immunoblot

Total protein extraction from AML cell lines was performed by directly incubating cells in SDS-containing lysis buffer at 95°C for 5 minutes followed by brief sonication to extract nuclear proteins. Total lysates were resolved by either 7.5% or 4-15% SDS-PAGE and transferred to a PVDF membrane (Biorad). Nonspecific binding was blocked in 5% non-fat milk for 1 hour at RT and blotted with the following antibodies: rabbit polyclonal anti-HIF-1α (Cayman) and mouse monoclonal anti-LGALS1 (C-8; Santa Cruz). Mouse anti-β-actin (Sigma) was used as internal loading control. When indicated, MOLM-13 cells (shCTRL or shHIF1-α) where treated for 16 hours with 200 μM CoCl_2_ before lysis. Protein quantification was performed using ImageJ software.

### Proliferation and apoptosis analysis

MOLM-13 cells (shCTRL and shHIF-1α) were plated at 2 × 10^5^ cells/mL into 12 well plates at day 0 and counted every day for 4 consecutive days using the Trypan blue exclusion method. Apoptosis was evaluated with Annexin V staining, performed using the PE Annexin V Apoptosis detection Kit I (BD Pharmingen) according to the manufacturer's protocol. Cell proliferation upon EZN-3088 and EZN-2968 electroporation was measured by Anti-Human Ki-67 set (BD Pharmingen) according to the manufacturer's instructions 24 hours upon electroporation.

### Flow cytometric analysis

For immunophenotypic analysis AML cell lines were plated at 4 × 10^5^ cells/mL into 6 well plates and, the day after, 1 × 10^6^ cells were collected and stained with the following fluorochrome-conjugated anti-human antibodies: CXCR4 (PE, R&D Systems) and CD18 (ITGB2, APC, BD Pharmingen). Staining was performed at 4°C for 20 minutes in the dark.

1 × 10^5^ events were acquired at flow cytometer (FACS Canto II, Becton Dickinson). Surface expression (percentage of positive cells) and MFI (mean fluorescent intensity) values were calculated for CXCR4 and ITGB2 with FlowJo software.

### Migration assays

1 × 10^6^ leukemic cells were seeded in the upper chamber of a transwell 6.5 mm diameter, 5 μm pore (Costar) w/wo 100 ng SDF-1α in the lower chamber. Migrated cells were recovered from the lower chamber 2.5 hours after seeding and counted by flow cytometer as the number of cells acquired per minute (FACS Canto II, Becton Dickinson). For migration experiments with EZN-3088 or EZN-2968 cells were seeded 24 hours after electroporation.

### Invasion assays

Invasive potential of MOLM-13 and THP-1 cells (shCTRL and shHIF-1α) was evaluated using BioCoat™ Matrigel Invasion Chamber (Corning). Matrigel coated inserts were rehydrated for 2 hours with RPMI 1640. 5 × 10^5^ cells were resuspended in medium supplemented with 0.1% FBS and added to the upper chamber, while lower chamber was supplemented with 15% FBS as chemoattractant. Cells that crossed the Matrigel-coated inserts were recovered from the lower compartments after 24 hours and counted by flow cytometer as the number of cells acquired per minute (FACS Canto II, Becton Dickinson).

### Transendothelial migration assays

HUVEC cells were seeded in the upper chamber of a transwell 6.5 mm diameter, 8 μm pore, (Costar) until complete confluence and formation of an endothelial monolayer barrier. The lower chamber was filled with 600 μL of cell culture media. 1.5 × 10^5^ MOLM-13 or THP-1 cells (shCTRL and shHIF-1α) were seeded on the surface of the endothelial monolayer. Cells that crossed the barrier were recovered from the lower chambers 24 hours after seeding and counted by flow cytometer as the number of cells acquired per minute (FACS Canto II, Becton Dickinson) to evaluated the transendothelial migration activity.

### Animal models

NOD/SCID/IL-2rγ^null^ (NSG) immunocompromised mice were maintained in a pathogen-free animal facility and treated in accordance with European Union guidelines. All animal protocols were approved by the Institutional Animal Care and Use Commitee (IACUC).

For homing experiments, mice were injected intravenously (i.v.) with 5 × 10^6^ MOLM-13 cells (shCTRL or shHIF-1α and EZN-3088 or EZN-2968) transduced with a bidirectional lentiviral vector co-expressing luciferase and truncated nerve growth factor receptor (ΔNGFR) and euthanized after 16 hours. Bone marrow (BM) samples were stained with anti-human CD271 (ΔNGFR) antibody (BD Pharmingen) and 5 × 10^6^ events acquired at flow cytometer (FACS Canto II, Becton Dickinson). For survival experiments, mice were challenged i.v. with 1 × 10^5^ MOLM-13 cells (shCTRL or shHIF-1α and EZN-3088 or EZN-2968) and sacrificed when terminally sick. Samples from BM, spleen and peripheral blood were stained with anti-human CD33 and anti-human CD271 (ΔNGFR) antibodies (BD Pharmingen) and acquired at flow cytometer (FACS Canto II, Becton Dickinson). To further investigate the colonization and dissemination of MOLM-13 cells (EZN-3088 and EZN-2968), NSG mice were injected i.v. with 1 × 10^5^ cells 24 hours after electroporation, and sacrificed at day 9 and day 15 after leukemia challenge. Samples from BM, spleen, peripheral blood, liver, kidneys and lungs were stained with anti-human CD33 and anti-human CD271 (ΔNGFR) antibodies (BD Pharmingen) and acquired at flow cytometer (FACS Canto II, Becton Dickinson). A separate cohort of animals was used for histopathological evaluation of organs colonization, when mice were sacrificed because terminally sick. Tissues were fixed in 4% formalin, paraffin embedded, cut into 5-μm thick sections, and stained with hematoxylin and eosin according to standard protocols. Sections were evaluated for leukemic dissemination by a certified pathologist.

### *In vivo* bioluminescence imaging

*In vivo* bioluminescence imaging (BLI) was performed by using the IVIS SpectrumCT System (Perkin Elmer). This system is equipped with a back-thinned, back-illuminated CCD camera cooled at −90°C with a quantum efficiency in the visible range above 85%.

Each animal received an intra-peritoneal injection of 150 mg luciferin/kg body weight 10 minutes before performing BLI. During BLI acquisition, the animals were kept at 37°C and under gaseous anesthesia (2–3% isoflurane and 1 l/min oxygen).

A set of images was acquired every 2 minutes from 10 to 20 minutes after luciferin injection in order to detect the highest BLI signal. The images were obtained using the following settings: exposure time=auto, binning=8, f=1 and a field of view equal to 13 cm (field C). Dark images were acquired before and then subtracted to bioluminescence images, no emission filters were used during BLI acquisitions.

### BLI image analysis

BLI image analysis was performed by placing region of interests (ROI) over the right and left posterior legs of the animals as shown in Supplementary Figure 3A. The total flux (photons/seconds) was measured in order to quantify light emission in each ROI. Images were acquired and analyzed using the Living Image 4.5 software (Perkin Elmer).

### Statistical analysis

Two-sided t-tests were used to validate the significance of the data analyzed. A *p* value of less than 0.05 was considered statistically significant. All analyses were performed with GraphPad Prism software (San Diego California). For survival experiments, curves were analyzed with the Mantel–Cox test.

## SUPPLEMENTARY MATERAILS FIGURES



## References

[1] Hasserjian RP (2013). Acute myeloid leukemia: advances in diagnosis and classification. Int J Lab Hematol.

[2] Pollyea DA, Kohrt HE, Medeiros BC (2011). Acute myeloid leukaemia in the elderly: a review. Br J Haematol.

[3] Bennett JM, Catovsky D, Daniel MT, Flandrin G, Galton DA, Gralnick HR, Sultan C (1976). Proposals for the classification of the acute leukaemias. French-American-British (FAB) co-operative group. Br J Haematol.

[4] Bennett JM, Catovsky D, Daniel MT, Flandrin G, Galton DA, Gralnick HR, Sultan C (1985). Proposed revised criteria for the classification of acute myeloid leukemia. A report of the French-American-British Cooperative Group. Ann Intern Med.

[5] Haferlach T, Schoch C, Schnittger S, Kern W, Loffler H, Hiddemann W (2002). Distinct genetic patterns can be identified in acute monoblastic and acute monocytic leukaemia (FAB AML M5a and M5b): a study of 124 patients. Br J Haematol.

[6] Villeneuve P, Kim DT, Xu W, Brandwein J, Chang H (2008). The morphological subcategories of acute monocytic leukemia (M5a and M5b) share similar immunophenotypic and cytogenetic features and clinical outcomes. Leuk Res.

[7] Peterson L, Dehner LP, Brunning RD (1981). Extramedullary masses as presenting features of acute monoblastic leukemia. Am J Clin Pathol.

[8] Porcu P, Cripe LD, Ng EW, Bhatia S, Danielson CM, Orazi A, McCarthy LJ (2000). Hyperleukocytic leukemias and leukostasis: a review of pathophysiology, clinical presentation and management. Leuk Lymphoma.

[9] Yan XJ, Xu J, Gu ZH, Pan CM, Lu G, Shen Y, Shi JY, Zhu YM, Tang L, Zhang XW, Liang WX, Mi JQ, Song HD, Li KQ, Chen Z, Chen SJ (2011). Exome sequencing identifies somatic mutations of DNA methyltransferase gene DNMT3A in acute monocytic leukemia. Nat Genet.

[10] Schoch C, Schnittger S, Klaus M, Kern W, Hiddemann W, Haferlach T (2003). AML with 11q23/MLL abnormalities as defined by the WHO classification: incidence, partner chromosomes, FAB subtype, age distribution, and prognostic impact in an unselected series of 1897 cytogenetically analyzed AML cases. Blood.

[11] Koh Y, Park J, Ahn KS, Kim I, Bang SM, Lee JH, Yoon SS, Soon Lee D, Yiul Lee Y, Park S, Kim BK (2009). Different clinical importance of FLT3 internal tandem duplications in AML according to FAB classification: possible existence of distinct leukemogenesis involving monocyte differentiation pathway. Ann Hematol.

[12] Boissel N, Renneville A, Biggio V, Philippe N, Thomas X, Cayuela JM, Terre C, Tigaud I, Castaigne S, Raffoux E, De Botton S, Fenaux P, Dombret H, Preudhomme C (2005). Prevalence, clinical profile, and prognosis of NPM mutations in AML with normal karyotype. Blood.

[13] Schlenk RF, Dohner K, Krauter J, Frohling S, Corbacioglu A, Bullinger L, Habdank M, Spath D, Morgan M, Benner A, Schlegelberger B, Heil G, Ganser A, Dohner H (2008). Mutations and treatment outcome in cytogenetically normal acute myeloid leukemia. N Engl J Med.

[14] Mrozek K, Marcucci G, Nicolet D, Maharry KS, Becker H, Whitman SP, Metzeler KH, Schwind S, Wu YZ, Kohlschmidt J, Pettenati MJ, Heerema NA, Block AW, Patil SR, Baer MR, Kolitz JE (2012). Prognostic significance of the European LeukemiaNet standardized system for reporting cytogenetic and molecular alterations in adults with acute myeloid leukemia. J Clin Oncol.

[15] Semenza GL (2013). HIF-1 mediates metabolic responses to intratumoral hypoxia and oncogenic mutations. J Clin Invest.

[16] Semenza GL (2012). Hypoxia-inducible factors: mediators of cancer progression and targets for cancer therapy. Trends Pharmacol Sci.

[17] Keith B, Johnson RS, Simon MC (2012). HIF1alpha and HIF2alpha: sibling rivalry in hypoxic tumour growth and progression. Nat Rev Cancer.

[18] Qing G, Simon MC (2009). Hypoxia inducible factor-2alpha: a critical mediator of aggressive tumor phenotypes. Curr Opin Genet Dev.

[19] Peng G, Liu Y (2015). Hypoxia-inducible factors in cancer stem cells and inflammation. Trends Pharmacol Sci.

[20] Wang Y, Liu Y, Malek SN, Zheng P (2011). Targeting HIF1alpha eliminates cancer stem cells in hematological malignancies. Cell Stem Cell.

[21] Zhang H, Li H, Xi HS, Li S (2012). HIF1alpha is required for survival maintenance of chronic myeloid leukemia stem cells. Blood.

[22] Yonekura S, Itoh M, Okuhashi Y, Takahashi Y, Ono A, Nara N, Tohda S (2013). Effects of the HIF1 inhibitor, echinomycin, on growth and NOTCH signalling in leukaemia cells. Anticancer Res.

[23] Rouault-Pierre K, Lopez-Onieva L, Foster K, Anjos-Afonso F, Lamrissi-Garcia I, Serrano-Sanchez M, Mitter R, Ivanovic Z, de Verneuil H, Gribben J, Taussig D, Rezvani HR, Mazurier F, Bonnet D (2013). HIF-2alpha protects human hematopoietic stem/progenitors and acute myeloid leukemic cells from apoptosis induced by endoplasmic reticulum stress. Cell Stem Cell.

[24] Coltella N, Percio S, Valsecchi R, Cuttano R, Guarnerio J, Ponzoni M, Pandolfi PP, Melillo G, Pattini L, Bernardi R (2014). HIF factors cooperate with PML-RARalpha to promote acute promyelocytic leukemia progression and relapse. EMBO Mol Med.

[25] Forristal CE, Brown AL, Helwani FM, Winkler IG, Nowlan B, Barbier V, Powell RJ, Engler GA, Diakiw SM, Zannettino AC, Martin S, Pattabiraman D, D'Andrea RJ, Lewis ID, Levesque JP (2015). Hypoxia inducible factor (HIF)-2alpha accelerates disease progression in mouse models of leukemia and lymphoma but is not a poor prognosis factor in human AML. Leukemia.

[26] Velasco-Hernandez T, Hyrenius-Wittsten A, Rehn M, Bryder D, Cammenga J (2014). HIF-1alpha can act as a tumor suppressor gene in murine acute myeloid leukemia. Blood.

[27] Vukovic M, Guitart AV, Sepulveda C, Villacreces A, O'Duibhir E, Panagopoulou TI, Ivens A, Menendez-Gonzalez J, Iglesias JM, Allen L, Glykofrydis F, Subramani C, Armesilla-Diaz A, Post AE, Schaak K, Gezer D (2015). Hif-1alpha and Hif-2alpha synergize to suppress AML development but are dispensable for disease maintenance. J Exp Med.

[28] Percio S, Coltella N, Grisanti S, Bernardi R, Pattini L (2014). A HIF-1 network reveals characteristics of epithelial-mesenchymal transition in acute promyelocytic leukemia. Genome Med.

[29] Coltella N, Valsecchi R, Ponente M, Ponzoni M, Bernardi R (2015). Synergistic Leukemia Eradication by Combined Treatment with Retinoic Acid and HIF Inhibition by EZN-2208 (PEG-SN38) in Preclinical Models of PML-RARalpha and PLZF-RARalpha-Driven Leukemia. Clin Cancer Res.

[30] Rouault-Pierre K, Hamilton A, Bonnet D (2015). Effect of hypoxia-inducible factors in normal and leukemic stem cell regulation and their potential therapeutic impact. Expert Opin Biol Ther.

[31] Harvey S, Zhang Y, Landry F, Miller C, Smith JW (2001). Insights into a plasma membrane signature. Physiol Genomics.

[32] Camby I, Belot N, Lefranc F, Sadeghi N, de Launoit Y, Kaltner H, Musette S, Darro F, Danguy A, Salmon I, Gabius HJ, Kiss R (2002). Galectin-1 modulates human glioblastoma cell migration into the brain through modifications to the actin cytoskeleton and levels of expression of small GTPases. J Neuropathol Exp Neurol.

[33] Abroun S, Otsuyama K, Shamsasenjan K, Islam A, Amin J, Iqbal MS, Gondo T, Asaoku H, Kawano MM (2008). Galectin-1 supports the survival of CD45RA(-) primary myeloma cells in vitro. Br J Haematol.

[34] Juszczynski P, Rodig SJ, Ouyang J, O'Donnell E, Takeyama K, Mlynarski W, Mycko K, Szczepanski T, Gaworczyk A, Krivtsov A, Faber J, Sinha AU, Rabinovich GA, Armstrong SA, Kutok JL, Shipp MA (2010). MLL-rearranged B lymphoblastic leukemias selectively express the immunoregulatory carbohydrate-binding protein galectin-1. Clin Cancer Res.

[35] Zhao XY, Zhao KW, Jiang Y, Zhao M, Chen GQ (2011). Synergistic induction of galectin-1 by CCAAT/enhancer binding protein alpha and hypoxia-inducible factor 1alpha and its role in differentiation of acute myeloid leukemic cells. J Biol Chem.

[36] Li ZH, Bresnick AR (2006). The S100A4 metastasis factor regulates cellular motility via a direct interaction with myosin-IIA. Cancer Res.

[37] Saleem M, Kweon MH, Johnson JJ, Adhami VM, Elcheva I, Khan N, Bin Hafeez B, Bhat KM, Sarfaraz S, Reagan-Shaw S, Spiegelman VS, Setaluri V, Mukhtar H (2006). S100A4 accelerates tumorigenesis and invasion of human prostate cancer through the transcriptional regulation of matrix metalloproteinase 9. Proc Natl Acad Sci U S A.

[38] Lo JF, Yu CC, Chiou SH, Huang CY, Jan CI, Lin SC, Liu CJ, Hu WY, Yu YH (2011). The epithelial-mesenchymal transition mediator S100A4 maintains cancer-initiating cells in head and neck cancers. Cancer Res.

[39] Wu JH, Tian XY, Hao CY (2011). The significance of a group of molecular markers and clinicopathological factors in identifying colorectal liver metastasis. Hepatogastroenterology.

[40] Glaser J, Neumann MH, Mei Q, Betz B, Seier N, Beyer I, Fehm T, Neubauer H, Niederacher D, Fleisch MC (2014). Macrophage capping protein CapG is a putative oncogene involved in migration and invasiveness in ovarian carcinoma. Biomed Res Int.

[41] Stefanidakis M, Karjalainen K, Jaalouk DE, Gahmberg CG, O'Brien S, Pasqualini R, Arap W, Koivunen E (2009). Role of leukemia cell invadosome in extramedullary infiltration. Blood.

[42] Matsunaga T, Takemoto N, Sato T, Takimoto R, Tanaka I, Fujimi A, Akiyama T, Kuroda H, Kawano Y, Kobune M, Kato J, Hirayama Y, Sakamaki S, Kohda K, Miyake K, Niitsu Y (2003). Interaction between leukemic-cell VLA-4 and stromal fibronectin is a decisive factor for minimal residual disease of acute myelogenous leukemia. Nat Med.

[43] Sun X, Cheng G, Hao M, Zheng J, Zhou X, Zhang J, Taichman RS, Pienta KJ, Wang J (2010). CXCL12 / CXCR4 / CXCR7 chemokine axis and cancer progression. Cancer Metastasis Rev.

[44] Viale A, De Franco F, Orleth A, Cambiaghi V, Giuliani V, Bossi D, Ronchini C, Ronzoni S, Muradore I, Monestiroli S, Gobbi A, Alcalay M, Minucci S, Pelicci PG (2009). Cell-cycle restriction limits DNA damage and maintains self-renewal of leukaemia stem cells. Nature.

[45] Gheldof A, Hulpiau P, van Roy F, De Craene B, Berx G (2012). Evolutionary functional analysis and molecular regulation of the ZEB transcription factors. Cell Mol Life Sci.

[46] Wellner U, Schubert J, Burk UC, Schmalhofer O, Zhu F, Sonntag A, Waldvogel B, Vannier C, Darling D, zur Hausen A, Brunton VG, Morton J, Sansom O, Schuler J, Stemmler MP, Herzberger C (2009). The EMT-activator ZEB1 promotes tumorigenicity by repressing stemness-inhibiting microRNAs. Nat Cell Biol.

[47] Brabletz S, Brabletz T (2010). The ZEB/miR-200 feedback loop–a motor of cellular plasticity in development and cancer?. EMBO Rep.

[48] Goossens S, Radaelli E, Blanchet O, Durinck K, Van der Meulen J, Peirs S, Taghon T, Tremblay CS, Costa M, Farhang Ghahremani M, De Medts J, Bartunkova S, Haigh K, Schwab C, Farla N, Pieters T (2015). ZEB2 drives immature T-cell lymphoblastic leukaemia development via enhanced tumour-initiating potential and IL-7 receptor signalling. Nat Commun.

[49] Valsecchi R, Coltella N, Belloni D, Ponente M, Ten Hacken E, Scielzo C, Scarfo L, Bertilaccio MT, Brambilla P, Lenti E, Martinelli Boneschi F, Brendolan A, Ferrero E, Ferrarini M, Ghia P, Tonon G (2016). HIF-1alpha regulates the interaction of chronic lymphocytic leukemia cells with the tumor microenvironment. Blood.

[50] Greenberger LM, Horak ID, Filpula D, Sapra P, Westergaard M, Frydenlund HF, Albaek C, Schroder H, Orum H (2008). A RNA antagonist of hypoxia-inducible factor-1alpha, EZN-2968, inhibits tumor cell growth. Mol Cancer Ther.

[51] Kiyoi H, Shiotsu Y, Ozeki K, Yamaji S, Kosugi H, Umehara H, Shimizu M, Arai H, Ishii K, Akinaga S, Naoe T (2007). A novel FLT3 inhibitor FI-700 selectively suppresses the growth of leukemia cells with FLT3 mutations. Clin Cancer Res.

[52] Tibshirani R, Hastie T, Narasimhan B, Chu G (2002). Diagnosis of multiple cancer types by shrunken centroids of gene expression. Proc Natl Acad Sci U S A.

[53] Huang da W, Sherman BT, Lempicki RA (2009). Bioinformatics enrichment tools: paths toward the comprehensive functional analysis of large gene lists. Nucleic Acids Res.

[54] Ferrero E, Ferrero ME, Pardi R, Zocchi MR (1995). The platelet endothelial cell adhesion molecule-1 (PECAM1) contributes to endothelial barrier function. FEBS Lett.

[55] Follenzi A, Ailles LE, Bakovic S, Geuna M, Naldini L (2000). Gene transfer by lentiviral vectors is limited by nuclear translocation and rescued by HIV-1 pol sequences. Nat Genet.

