# Supplementary material for: Hypoxia inducible factor-1α regulates a pro-invasive phenotype in acute monocytic leukemia
Source: Oncotarget. 2016 Jul 18;7(33):53540–57. doi: 10.18632/oncotarget.10660 (PMC5288204; doi:10.18632/oncotarget.10660)
Supplement: Supplementary file 1 [file oncotarget-07-53540-s001.pdf]

Hypoxia inducible factor-1α regulates a pro-invasive phenotype in acute monocytic leukemia

SUPPLEMENTARY FIGURES

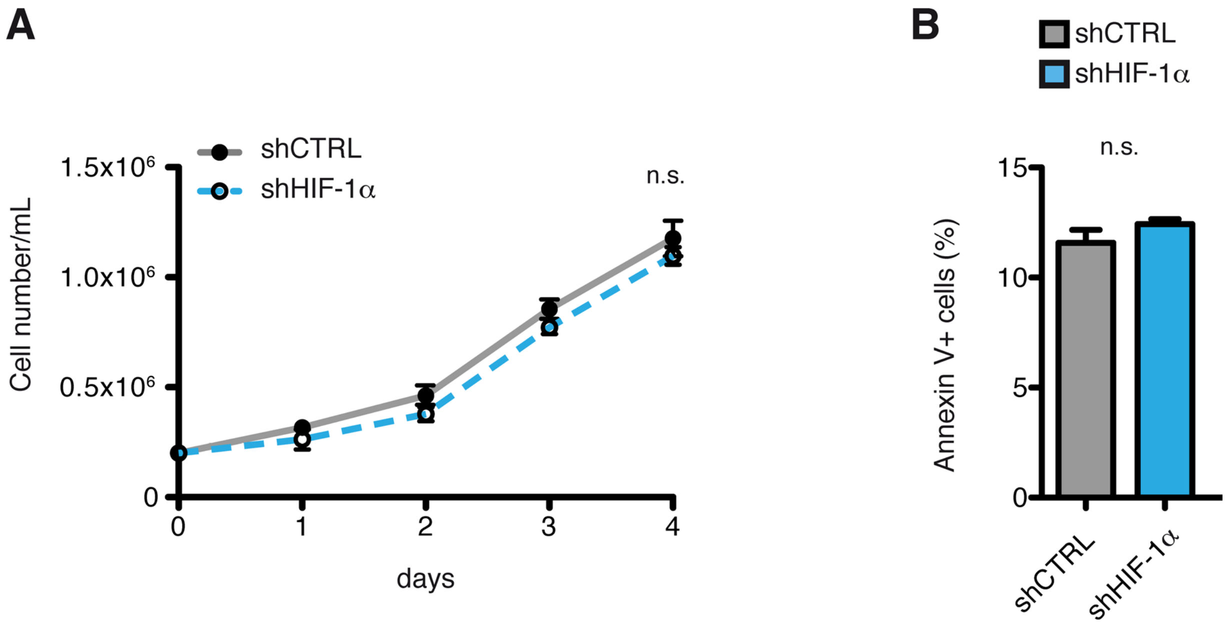

Supplementary Figure S1: HIF-1α chronic silencing does not affect cell proliferation and apoptosis of MOLM-13 cells *in vitro*. A. Proliferation curve of shCTRL and shHIF-1α MOLM-13 cells. Graph represents cell number/mL. Data represent mean values ± s.e.m. of three independent experiments. **B.** Percentage of Annexin V$^+$ shCTRL and shHIF-1α MOLM-13 cells. Graph represents mean values ± s.e.m. of three independent experiments.

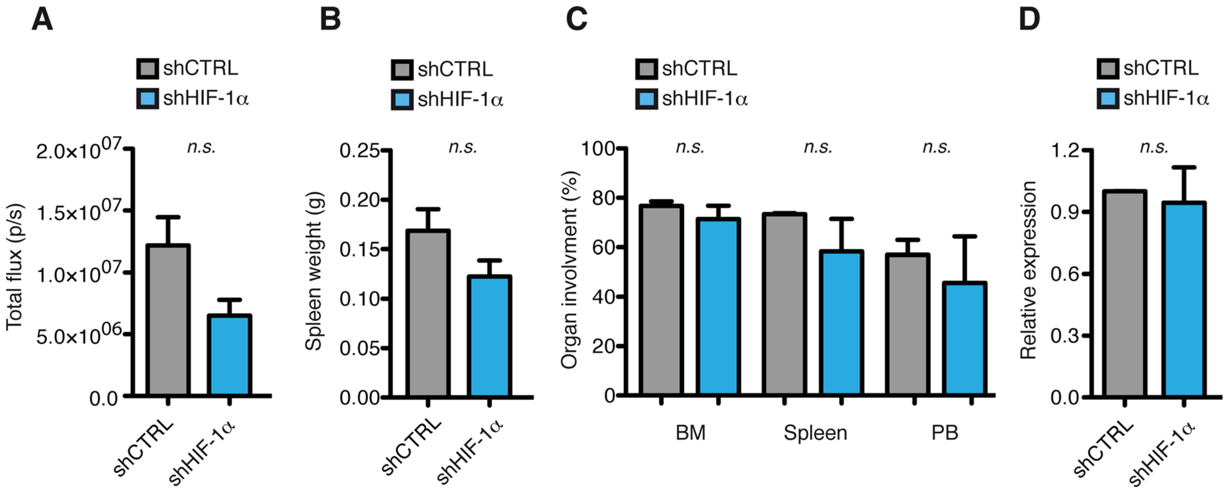

Supplementary Figure S2: HIF-1α silencing is lost in long term *in vivo* experiments. **A.** Quantified light output expressed as total flux (photons/seconds) from each ROI drawn on the right and left posterior legs of mice injected with MOLM-13 shCTRL and shHIF-1α and co-expressing ΔNGFR and luciferase 9 days post leukemia challenge (n=3 for each group). **B.** Spleen weight of mice injected with MOLM-13 shCTRL and shHIF-1α and sacrificed when terminally sick (n=3 for each group). **C.** Organ involvement expressed as percentage of $CD33^+$ $\Delta NGFR^+$ human cells in different organs from mice transplanted and sacrificed as in (B) (n=3 for each group). **D.** Real-time PCR analysis of HIF-1α in the BM of mice transplanted and sacrificed as in (B). Data represent mean values ± s.e.m. of 3 mice.

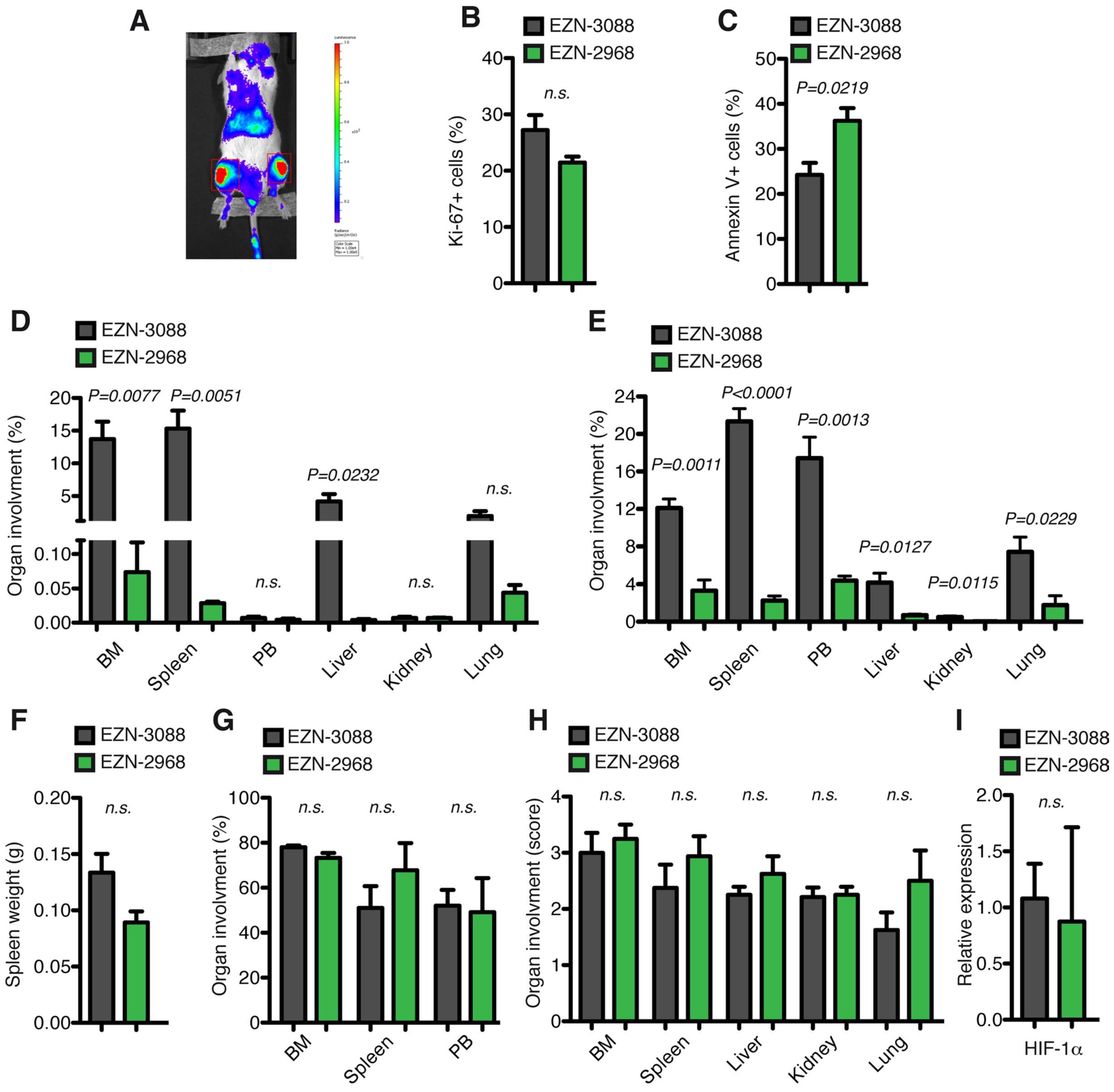

Supplementary Figure S3: HIF-1α inhibition is lost in long term *in vivo* experiments. A. Image of ROI drawn on the right and left posterior legs of a representative mouse injected with MOLM-13 cells transfected with EZN-3088 and co-expressing ΔNGFR and luciferase 5 days post leukemia challenge. **B.** Percentage of Ki-67^{+} EZN-3088 and EZN-2968 MOLM-13 cells 24 h after transfection. **C.** Percentage of Annexin V^{+} EZN-3088 and EZN-2968 MOLM-13 cells 24 h after transfection. (B-C) Data represent mean values ± s.e.m. of three independent experiments. **D-E.** Organ involvement expressed as percentage of CD33^{+} ΔNGFR^{+} human cells in different organs from mice injected with MOLM-13 cells transfected with EZN-3088 or EZN-2968 and co-expressing ΔNGFR and luciferase. Mice were sacrificed at day 9 (D) or at day 15 (E) post leukemia challenge (n=4 for each group). **F.** Spleen weight of mice injected with MOLM-13 cells transfected as in (D) and sacrificed when terminally sick (n=4 for each group). **G.** Organ involvement expressed as percentage of CD33^{+} ΔNGFR^{+} human cells in different organs from mice transplanted and sacrificed as in (F) (n=4 for each group). **H.** Organ involvement expressed as histopathological score of organs colonization in mice transplanted and sacrificed as in (F) (n=4 for each group). **I.** Real-time PCR analysis of HIF-1α in the BM of mice transplanted and sacrificed as in (F). Data represent mean values ± s.e.m. of 3 mice.
